# Co-occurrence of behavioural risk factors for non-communicable diseases among 40-year and above aged community members in three regions of Myanmar

**DOI:** 10.12688/openreseurope.15859.1

**Published:** 2023-05-15

**Authors:** Aye Sandar Mon, Hla Hla Win, Win Pa Sandar, Poppy Walton, Khin Hnin Swe, Johanna P. M. Vervoort, Jeanet A. Landsman, Martin Rusnak, Jaap A. R. Koot

**Affiliations:** 1 Department of Biostatistics and Medical Demography, University of Public Health, Yangon, Yangon, Yangon, 11011, Myanmar; 2 University of Public Health, Yangon, Yangon, Yangon, 11011, Myanmar; 3 SUNI-SEA project, HelpAge International Myanmar, Yangon, Yangon, 11011, Myanmar; 4 Global Health Unit, Department of Health Sciences, University Medical Center Groningen, Groningen, 9713 AV, The Netherlands; 5 Department of Health Science, University Medical Center Groningen, Groningen, 9700 RB, The Netherlands; 6 Department of Public Health, Faculty of Health Care and Social Work, University of Trnava, Trnava, Slovakia

**Keywords:** behavioural risk factors, risk behaviours, multiple risk factors, co-occurrence, NCD

## Abstract

**Background:** Risky behaviours such as smoking, alcohol consumption, physical inactivity and inadequate consumption of fruits and vegetables are known contributing factors for non-communicable diseases (NCDs) which account for 74% of global mortality. Such behavioural risk factors co-occur frequently resulting in synergistic action for developing NCD related morbidity and mortality. This study aims to assess the existence of multiple risk behaviours and determine the socio-economic and demographic factors associated with co-occurrence of behavioural risks among Myanmar adult population.

**Method:** Data were collected, in the context of the SUNI-SEA project (Scaling Up NCD interventions in Southeast Asia), from 660 community members aged 40 years and above of both sexes, residing in selected urban and rural areas from Ayeyawaddy, Yangon and Mandalay regions of Myanmar. The co-occurrence of behavioural risk factors was presented as percentage with 95% CI and its determinants were identified by multinomial logistic regression.

**Results:** The co-occurrence of two risk behaviours and three or four risk behaviours were found in 40% (95% CI: 36.2%, 43.9%) and 10.8% (95% CI: 8.5%, 13.4%) respectively. Urban residents, men, participants without formal schooling and unemployed persons were more likely to exhibit co-occurrence of two risk behaviors and three or four risk behaviours.

**Conclusion:** The current study shows high prevalence of co-occurrence of behavioural risk factors among Myanmar adults in the study area. NCD prevention and control programs emphasizing management of behavioural risks should be intensively promoted, particularly directed towards multiple behavioural risk factors, and not focused on individual factors only.

## Plain language summary

Risky behaviours such as smoking, alcohol consumption, physical inactivity and inadequate consumption of fruits and vegetables are known contributing factors for non-communicable diseases (NCDs) which account for 74% of global mortality. Such behavioural risk factors co-occur frequently resulting in synergistic action for developing NCD related morbidity and mortality. This study aims to assess the existence of multiple risk behaviours and determine the socio-economic and demographic factors associated with co-occurrence of behavioural risks among the Myanmar adult population. Data were collected from 660 community members aged 40 years and above of both sexes, residing in selected urban and rural areas from Ayeyawaddy, Yangon and Mandalay Regions of Myanmar. The current study shows substantial proportion of study adults had co-occurrence of behavioural risk factors (at least two risk factors), contributing to 50.8%. Urban residents, men, participants without formal schooling and unemployed persons were more likely to present co-occurrence of two risk behaviors and three or four risk behaviours, compared with their counterparts. Based on the study findings, the research team highly recommends that NCD prevention and control programs emphasizing management of behavioural risks should be intensively promoted, particularly directed towards multiple behavioural risk factors, and not focused on single risk factor only.

## Introduction

Non-communicable diseases (NCDs) have become the major public health burden across the world, also in low- and middle-income countries (LMIC) since NCDs have been the leading causes of morbidity and mortality in the past three decades ^1, 2^. The World Health Organization (WHO) estimates that NCDs account for 74% of global mortality, and in Myanmar, NCDs accounted for 59% of total deaths in 2014, which increased to an alarming 68% in 2018 ^3^. NCDs such as cardiovascular diseases, diabetes and cancer share major behavioural risks such as smoking, excessive alcohol consumption, unhealthy diet and physical inactivity. Those risk factors can cluster and interact resulting in synergistic action for developing NCDs and NCD related mortality ^4^. Kaukua, *et al.* provided evidence that the incidence of first myocardial infarction increased from 0% to 40% with the increased number of co-occurrences of risk factors from zero to five ^5^. Moreover, a large prospective cohort study conducted in United Kingdom revealed that individuals who experienced all four major behavioural risk factors, such as smoking, high levels of alcohol consumption, unhealthy diet and physical inactivity, had about three times the risk of cardiovascular disease (CVD) and cancer mortality compared to those experiencing none of the risks. Meanwhile, it was found that this cause-specific mortality was 1.9 times higher for individuals with one risk, about two times for those with two risks, around 2.8 times for those with three risks and 3.5 times for those with four risks ^6^. Therefore, evidence indicates that persons who engage in multiple behavioural risk factors are likely to have significantly worse health outcomes than those engaging in one health risk behaviour ^7^.

Such behavioral risks co-occur frequently, resulting in accumulating risks. A number of international studies revealed that more than half of the study adults were found to engage in two or more behavioural risks ^8^. According to Myanmar 2014 STEP survey report, among the 45 to 64-year aged group, the prevalence of co-occurrence of three or more risk factors was 29.3%. In that Myanmar STEP survey, biological and metabolic risk factors such as obesity and raised blood pressure in addition to behavioural risk factors were considered a combined risk ^9^.

While co-occurrence of those risk factors impacts on NCD related morbidity and mortality, adopting healthy lifestyle behaviour can correspondingly reduce the risk of NCDs impacting other NCDs, as well. Behavioural risks are considered modifiable factors that are more sensitive to interventions than biological and metabolic risk factors ^10^. Understanding the co-occurrence of NCD risk factors can provide the information required for better prevention and management strategies in order to mitigate NCD-related morbidity and premature death.

Socioeconomic and demographic factors such as age, gender, residence, occupation, education, income and health literacy of individuals play an important role in shaping the practice of lifestyle behaviour. An increasing number of international studies have explored the evidence concerning the impact of socioeconomic variables as predictors of multiple risk behaviours ^7, 8, 11^. However, to our knowledge, there is still scarcity of such evidence for Myanmar adults, specifically from remote communities. This study aims to assess the existence of multiple risk behaviours and determine the socio-economic and demographic factors associated with co-occurrence of behavioural risks among study Myanmar adult population.

## Methods

### Study design

For this study, the cross-sectional survey data collected in the baseline measurement from the retrospective phase of the SUNI-SEA project was used. “Scaling-Up NCD Interventions in South-East Asia (SUNI-SEA)” is a research project in which nine consortium members from South-East Asia and Europe collaborate. The project aims to strengthen the provision of diabetes and hypertension prevention, and management services through evidence-based research in Indonesia, Myanmar and Vietnam, by better understanding effective scaling up strategies for existing NCD interventions ^12^.

### Study population

The study population consists of community members aged 40 years and above of both sexes, residing in selected urban and rural areas from Ayeyawaddy, Yangon and Mandalay regions of Myanmar.

### Sample size and sampling design

Participants were selected using multistage sampling. Firstly, six townships were selected from Ayeyawaddy, Yangon and Mandalay Regions (two townships from each region), as those townships had the most concentrated numbers of wards or village tracts/villages that had an inclusive self-help group (ISHG), which is a community club that offers activities or benefits across multiple domains: health, income security and social integration. These include various activities such as basic health checks where primary care services are not easily accessible, limited financial assistance with the costs of care, including transport costs in emergencies, health education sessions, and home visitation and care, especially for the home bound. In each selected township, two clusters (ward or village) were selected using stratified random sampling based on the presence or absence of ISHGs. The sampling frame required for first and second stages of sampling procedures was based on the ISHG data from HelpAge International. Subsequently, from each of the clusters, 55 households having at least one 40-year-aged household member were selected using the random walk method. Finally, one eligible household member was recruited from each selected household, totaling 660 participants. If there was more than one eligible member in the selected household, then one member was selected randomly using a lottery method.

### Data collection procedure

Data collection was undertaken during January 2020 using a structured questionnaire which included information on background characteristics of participants, lifestyle factors, knowledge and practice related to hypertension and diabetes mellitus, and knowledge and practice regarding healthcare mobilization services provided by ISHG. For data collection, in order to minimize the inter-interviewer variability and interviewer bias, seven interviewers were trained for two days. Pretesting of the questionnaire was carried out in a selected township of Yangon, which was not included in the study area. Face-to-face interviews were conducted using the pretested and structured questionnaire ^13, 14^ by Open Data Kit (ODK) software.

### Variables

***Outcome variable***. The outcome variable, co-occurrence of behavioural risk factors, was treated as three-level categorical variable categorizing (1) no risk or single risk (2) co-occurrence of two risks (3) co-occurrence of three or four risks. The behavioural risk factors for NCDs in this study were considered smoking, alcohol consumption, insufficient physical activity and inadequate consumption of fruits and vegetables.

***Independent variables***. The socio-demographic variables, age, sex, marital status, education, employment status, ISHG membership and knowledge of risk factors of hypertension and diabetes are considered independent variables.

### Operational definitions

Operational definitions, for data categorization and analysis, based on sources such as WHO, were made as following: (1) smoking was considered when anyone currently smokes any tobacco products; (2) alcohol consumption was defined as consuming alcoholic drinks over the past 30 days; (3) insufficient physical activity was defined as less than 150 minutes of moderate physical activity, or less than 70 minutes of intense physical activity, or less than 150 minutes of a combination of both, weekly; (4) inadequate consumption of fruits and vegetables was defined as eating less than five servings of fruit and/or vegetables on average per day ^15^.

### Statistical analysis

Data extraction, exploration and analysis were conducted using Stata 15.1. The proportions of each level of co-occurrence of behavioural risks by age and sex structure were mentioned together with 95% confidence interval (CI). To determine the factors associated with co-occurrence of behavioural risks, bivariate and multivariable analyses were done. The bivariate analysis using Chi-squared test was performed to select the explanatory variables for the multivariable model. As the outcome variable was a three-level categorical variable, the multinomial logistic regression model was applied for multivariable analysis. The sociodemographic variables that had a p-value less than 0.3 in bivariate analysis were considered explanatory variables for the multivariable model. The relative risk ratio (RRR) together with 95% CI was presented as the effect estimate for explanatory variables on outcome variable. Assumption for multi-collinearity among explanatory variables was checked and there was no collinearity issue. After running the multivariable model, post logistic regression diagnostic tests such as model fitness and model specification were also checked, and the final model passed those tests. All test statistics were treated as two-sided, and level of significance was set at 5%.

This study was conducted according to the guidelines of the Declaration of Helsinki and approved by the Institutional Review Board of Department of Medical Research in Myanmar (protocol code – Ethics/DMR/2019/145).

Before interviewing the study participants, the purpose and detailed procedures of the study and benefits and risks of participation were explained to participants and written informed consent to participate was obtained from all participants.

## Results

In this study, most of the participants were from rural areas (61.5%) and the majority were females (72.9%). The mean (SD) age of respondents was 57.5 (10.7) years and their age ranged from 40 to 87 years.

### Co-occurrence of behavioural risk factors for NCD by age and sex structure

The distribution of co-occurrence of behavioural risk factors for NCD according to age and sex structure was presented in Table 1. Among 660 participants, 49.2% had no risk or single behavioural risk, 40% had two risks simultaneously and 10.8% had three or four risks together. The percentage of respondents having three or four risks simultaneously became higher with increasing age in females, but this percentage was highest in 50–59 years aged group among males.

**Table 1.** Distribution of co-occurrence of behavioural risk factors for NCD by age and sex structure.

Variables	Total frequency	Co-occurrence of behavioural risk factors for NCD					
		No risk or single risk		Two risks		Three or more risks	
		%	95%CI	%	95%CI	%	95%CI
**Males** **(N=179)**							
Age groups							
40–49	34	35.3	19.7, 53.5	35.3	19.7, 53.5	29.4	15.1, 47.5
50–59	48	35.4	22.2, 50.5	33.3	20.4, 48.4	31.3	18.7, 46.3
60–69	61	36.1	24.1, 49.4	47.5	34.6, 60.7	16.4	8.2, 28.1
70+	36	38.9	23.1, 56.5	44.4	27.9, 61.9	16.7	6,4, 32.8
**Females (N=481)**							
Age groups							
40–49	140	60.7	52.1, 68.9	37.9	29.8, 46.4	1.4	0.2, 5.1
50–59	162	58.0	50.0, 65.7	37.0	29.6, 45.0	4.9	2.2, 9.5
60–69	119	51.3	41.9, 60.5	39.5	30.7, 48.9	9.2	4.7, 15.9
70+	60	33.3	21.7, 46.7	51.7	38.4, 64.8	15.0	7.1, 26.6
**Total (N=660)**							
Age groups							
40–49	174	55.7	48.0, 63.3	37.4	30.2, 45.0	6.9	3.6, 11.7
50–59	210	52.9	45.9, 59.8	36.2	29.7, 43.1	11.0	7.1, 16.0
60–69	180	46.1	38.7, 53.7	42.2	34.9, 49.8	11.7	7.4, 17.3
70+	96	35.4	25.9, 45.8	49.0	38.6, 59.4	15.6	9.0, 24.5
**Overall**	**660**	**49.2**	**45.4, 53.1**	**40**	**36.2, 43.9**	**10.8**	**8.5, 13.4**

**Figure 1.**
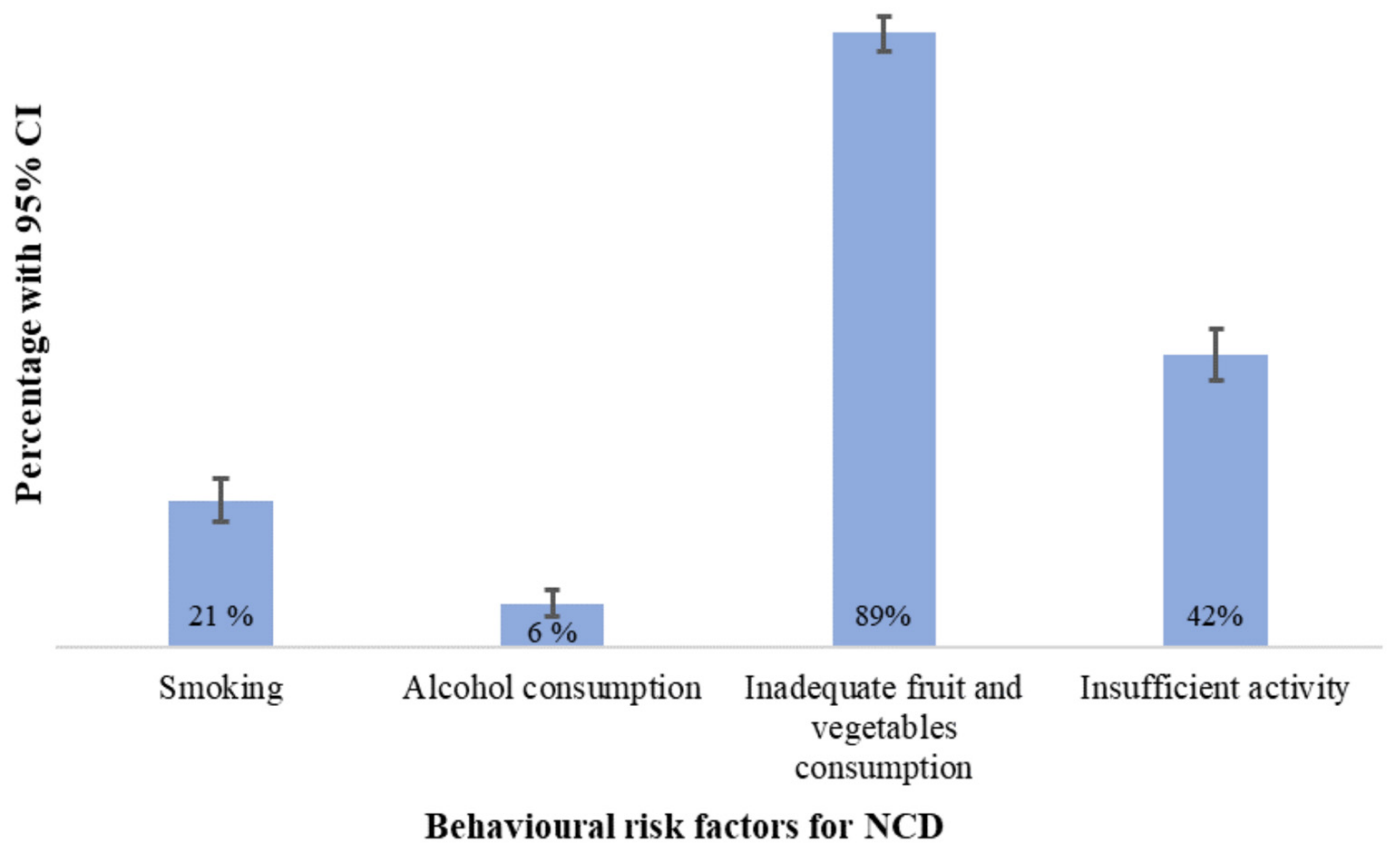
Distribution of behavioural risk factors for NCD.

Among the four risk behaviours, inadequate consumption of fruit and vegetables was the most frequent risk factor, exhibited by 89% (95% CI: 87%, 92 %) of participants, followed by insufficient physical activity, 42% (95% CI: 39%, 46%). Twenty-one percent (95% CI: 18%, 25%) were smokers while six percent (95% CI: 4%, 8%) reported they consumed alcohol ( Figure 1).

### Distribution of behavioural risk factors by socio-demographic characteristics

According to Table 2, in assessing sociodemographic differentials of each behavioural risk factor for NCD, rural participants had higher percentages for smoking, alcohol consumption and inadequate consumption of fruits and vegetables but lower percentages for insufficient physical activity. Males had higher percentages of smoking and alcohol consumption while females had higher percentages of inadequate fruit and vegetables consumption and insufficient physical activity. The participants having good knowledge on risk factors for hypertension and diabetes (above median) presented lower percentages for all four behavioural risk factors for NCD compared to those having low knowledge level (median and below).

**Table 2.** Distribution of each behavioural risk factor according to socio-demographic characteristics of respondents.

Variables	Total frequency	Smoking	Alcohol consumption	Inadequate fruit and vegetables consumption	Insufficient physical activity
		%	%	%	%
Residence					
Urban	254	15.0	5.9	88.3	50.8
Rural	406	25.1	6.4	89.9	37.2
Age groups					
40–49	174	10.3	7.5	84.2	40.8
50–59	210	22.9	7.1	89.9	37.1
60–69	180	27.2	6.1	90.7	40.0
70+	96	26.0	2.1	94.4	61.5
Gender					
Male	179	37.4	21.8	88.3	36.3
Female	481	15.2	0.4	89.6	44.7
Marital status					
Never married	27	25.9	7.4	92.0	33.3
Currently married	470	20.4	7.2	89.0	41.1
Separated/ widow/ divorcee	161	22.9	3.1	89.3	47.2
Education level					
No formal schooling	165	30.9	1.2	89.6	46.7
Primary	285	20.4	6.3	91.3	40.0
Secondary and above	209	14.8	10.1	86.4	42.1
Occupation					
Employed	337	23.2	8.9	86.8	29.9
Unpaid work	193	16.6	2.6	90.1	47.7
Unemployed	130	23.1	4.6	90.3	66.9
Knowledge on risk factors for hypertension & diabetes mellitus					
Median & below	371	23.2	7.0	90.4	44.5
Above median	289	18.7	5.2	87.9	39.8
Being ISHG member					
No	558	20.6	7.0	89.1	43.9
Yes	102	24.5	2.0	89.9	34.3

### Socio-demographic Characteristics and Co-occurrence of Behavioural Risk Factors

***Bivariate Analysis using Chi-squared Test***. Table 3 revealed the results from bivariate analysis using Chi-squared test in which the outcome variable was co-occurrence of behavioural risk factors for NCD, which was three-level categorical variable; (1) no behavioural risk factor or single risk, (2) co-occurrence of two risks simultaneously and (3) three or four risks simultaneously. The independent variables were the sociodemographic characteristics of respondents such as residence, age group, gender, marital status, education, occupation, knowledge on risk factors for hypertension and diabetes and being ISHG member and then all were considered categorical variables. According to this table, statistically significant associations were observed between the outcome variable and the residence, age group, gender and occupation. Rural residents had a lower proportion of co-occurrence of two risk factors but a higher proportion of co-occurrence of three or four risks compared to urban residents. Moreover, the proportion of co-occurrence of three or four behavioural risk factors increased with age, was higher in males compared to females, and was highest among unemployed participants compared to those having unpaid work and those having income generating employment. Although there was no significant association between the outcome and other socio-demographic variables such as marital status, education, knowledge on risk factors and being ISHG member, the proportion of co-occurrence of three or four risks was higher among the participants having no formal schooling than among those with primary school level and those with secondary or above education. Moreover, the participants having above median knowledge level and ISHG members had lower percentage of co-occurrence of two risks as well as three or four risks than their respective counterparts.

**Table 3.** Association between socio-demographic characteristics and co-occurrence of behavioural risk factors (Bivariate analysis using Chi-squared test).

Variables	Total frequency	Co-occurrence of behavioural risk factors			*P*-value
		No risk or single risk	Two risks	Three or more risks	
		%	%	%	
Residence					**0.015**
Urban	254	44.5	46.8	8.7	
Rural	406	52.2	35.7	12.0	
Age groups					**0.029**
40–49	174	55.7	37.4	6.9	
50–59	210	52.8	36.2	11.0	
60–69	180	46.1	42.2	11.7	
70+	96	35.4	49.0	15.6	
Gender					**<0.001**
Male	179	36.3	40.8	22.9	
Female	481	54.1	39.7	6.2	
Marital status					0.997
Never married	27	48.2	40.7	11.1	
Currently married	470	49.8	39.4	10.8	
Separated/ widow/ divorcee	161	48.5	41.0	10.5	
Education level					0.218
No formal schooling	165	45.4	39.4	15.2	
Primary	285	50.2	41.4	8.4	
Secondary and above	209	51.2	38.3	10.5	
Occupation					**<0.001**
Employed	337	56.7	31.7	11.6	
Unpaid work	193	48.7	44.6	6.7	
Unemployed	130	30.8	54.6	14.6	
Knowledge on risk factor for hypertension & diabetes mellitus					0.173
Median & below	371	46.1	42.9	11.0	
Above median	289	53.3	36.3	10.4	
Being ISHG member					0.654
Non-member	558	48.6	40.3	11.1	
Member	102	52.9	38.3	8.8	

***Multivariable Analysis using Multinomial Logistic Regression.*** The results from multinomial logistic regression are outlined in Table 4. In this multivariable model, the outcome variable was co-occurrence of behavioural risk factors for NCD, three-level categorical variable. There were six explanatory variables which were selected from those having p-value less than 0.3 in bivariate analysis: residence (urban-rural), age group, gender, educational level, occupation of respondents and knowledge on risk factors for hypertension and diabetes. The age group variable was treated as a binary variable (only two-level) to reduce the number of parameters that were estimated from the model and minimize the model complexity issue. Statistical tests for checking assumptions such as collinearity, and tests for model fitness were also performed and the analysis passed those tests. The study participants from rural areas were less likely to co-occur two behavioural risk factors relative to occurrence of no risk or single risk by 35% compared to those from urban areas. In comparison to males, the co-occurrence of two risks significantly reduced among females (RRR: 0.53, 95%CI: 0.35, 0.82) and consistent findings were observed for co-occurrence of three or four risks relative to no risk or single risk (RRR: 0.15, 95%CI: 0.08, 0.28). The participants belonging to the group that has a primary education level had 52% lower odds of presenting three or four behavioural risk factors simultaneously and those with secondary or above level had 60% lower odds of co-occurrence of three or four risks than those without formal schooling. Comparing the participants in employment, the unemployed participants had a relative risk ratio of 2.94 (95%CI: 1.76, 4.88) for co-occurrence of two risks and 2.42 (95%CI: 1.14, 5.15) for co-occurrence of three or more risks. Participants with unpaid work including homemakers and retired persons had a significantly higher relative risk ratio for co-occurrence of two risk factors (RRR: 1.58, 95%CI: 1.04, 2.41) relative to no risk or single risk.

**Table 4.** Multinomial logistic regression for co-occurrence of behavioural risk factors for NCD.

Variables	No risk/ Single risk (base) *vs* Two risks		No risk/ Single risk (base) *vs* Three or more risks	
	RRR	95%CI	RRR	95%CI
Residence	*P* **=0.039**		*P*=0.566	
Urban	1		1	
Rural	0.65	0.43, 0.98	0.82	0.41, 1.63
Age groups	*P*=0.775		*P*=0.882	
< 60 years	1		1	
60+	1.05	0.72, 1.55	0.96	0.52, 1.75
Gender	*P* **=0.004**		*P* **<0.001**	
Male	1		1	
Female	0.53	0.35, 0.82	0.15	0.08, 0.28
Education level	*P*=0.191		*P* **=0.050**	
No formal schooling	1		1	
Primary	1.09	0.69, 1.71	0.48	0.24, 0.96
Secondary or above	0.73	0.42, 1.26	0.40	0.17, 0.90
Occupation	*P* **=0.0002**		*P* **=0.039**	
Employed	1		1	
Unpaid work	1.58	1.04, 2.41	0.97	0.45, 2.08
Unemployed	2.94	1.76, 4.88	2.42	1.14, 5.15
Knowledge on risk factor for hypertension & diabetes mellitus	*P*=0.347		*P*=0.949	
Median & below	1		1	
Above median	0.85	0.60, 1.20	1.02	0.58, 1.78

## Discussion

This study was conducted to assess the existence of multiple risk behaviours for NCD and its related socio-demographic factors among Myanmar adults. The study findings revealed that, among four behavioural risk factors for NCD, inadequate consumption of fruits and vegetables was the most frequent risk factor. Nearly 90% of the study participants consumed less than five servings of fruits and vegetables on average per day. Regarding insufficient physical activity, the second most common behavioural risk in this study, two in five did not meet the WHO recommendation on physical activity for health. Concerning smoking and alcohol consumption, one-fifth of the study participants were smokers while only 6% reported they consumed alcohol. These findings for inadequate consumption of fruits and vegetables and smoking were similar to those from the Myanmar STEP survey 2014, in which 87% of participants had inadequate fruit and vegetables consumption and 21% were daily smokers. However, for alcohol consumption and physical inactivity, the evidence of this study was different from the STEP survey 2014. The prevalence of alcohol consumption was lower than in the STEP survey (6% vs 19.8%) while the proportion of insufficient physical activity was clearly higher than in the STEP survey (42% vs 15.7%). These discrepancies may occur due to the difference in age composition of study participants. Over one-third of the participants were 60-year and above in this study, whilst the maximum age of study participants in the STEP survey was 64 years ^9^. The distinctively high proportion of inadequate consumption of fruits and vegetables was comparable with the results from studies conducted in West Bengal, Kenya and Ethiopia, where this behavioural risk was the most common in comparison to the other three behavioural risks ^16– 18^. This similarity may be due to the similar socio-economic status of the countries where the studies were conducted, which are all developing countries. Socio-economic status then may be connected to access to the daily fruit and vegetables requirements due to financial difficulty.

The present study highlighted that half of study participants had at least two behavioural risk factors simultaneously (40% for co-occurrence of two risks and 10.8% for three or more risks). This substantial number of study participants with multiple unhealthy lifestyle behaviours indicates that there is a need for public health interventions focusing on the prevention of NCD to avert future unfavourable consequences. Similar evidence of co-occurrence of two or more behavioural risk factors has been observed in a study conducted in Uganda, where 56.4% had a combination of risk factors ^19^. However, the prevalence of co-occurrence of at least two risks of the current study was higher than that of a Brazilian study where 37% had co-occurrence of two or more risks and smoking, abusive use of alcohol, unhealthy eating, physical inactivity during leisure time and obesity were considered the risk factors. This inconsistency may be due to the heterogeneity in defining the behavioural risk variables ^20^. Meanwhile, the extent of this co-occurrence was lower compared with an Ethiopian study in which 65.5% had co-occurrence despite both studies using the same operational definitions in assessing the risk variables ^18^. The possible explanation may be due to dissimilarity in study settings. The current study was carried out in both urban and rural areas, but the Ethiopian study was conducted only in an urban setting.

Based on the results from the multivariable model performed in this study, residence, gender, educational level and occupation of the study participants were observed as the factors that were significantly related to co-occurrence of behavioural risk factors. Participants residing in urban areas were more likely to co-occur at least two risk factors compared to rural residents. Similar findings were also reported in other studies conducted in Uganda and Bhutan ^19, 21^. Urban residents are more prone to be physically inactive than rural residents due to the consequences of urbanization such as easy access to transportation leading to lesser walking activity, sedentary working conditions, and inactive types of recreation. This indicates that there is a need for public health programs that encourage physical activity and that these are particularly necessary in urban areas. In this study, the rising pattern of co-occurrence with increasing age was observed for existence of two risk factors as well as three or more risk factors, although age was not found out as a significant predictor in the multivariable model. This seems consistent with evidence from two Kenyan studies ^17, 22^. Assessing gender difference in simultaneous occurrence of multiple risk factors, male participants had a greater tendency of having co-occurrence of two risk factors as well as three or more risks than females. This finding was consistent with other studies from Brazil, Kenya and Chile ^10, 20, 22, 23^. Two systematic reviews also supported this evidence ^8, 24^. The two important behavioural risks for NCD, smoking and alcohol consumption, cumulatively and more frequently occur among men may cause exhibiting co-occurrence of behavioural risks in males. Regarding the association between educational level and co-occurrence of risks, participants without formal schooling were more likely to present three or more behavioural risks than those with formal schooling. Consistently, two systematic reviews reported similar results in which lower educational levels had a strong association with the co-occurrence of multiple risk factors ^8, 24^. The potential reason behind this may be that persons with higher education attainment are more likely to seek health information about NCDs and their risk factors from reliable sources of information.

 Differences in employment status existed across the co-occurrence of risk behaviours as the unemployed participants had nearly three times the odds of co-occurrence of two risk factors and 2.5 times odds of exhibiting three or more risk factors compared with currently employed participants. The evidence was supported by two studies conducted in Ethiopia and Kenya in which unemployed participants were more likely to co-occur at least two risk factors than employed ones ^18, 23^. Though there were no significant associations between being ISHG member, presence of knowledge on hypertension and diabetes and the co-occurrence of behavioural risk factors, higher proportions of co-occurrence of two risks as well as three/more risks were observed among ISHG members and the participants having knowledge compared with their counterparts respectively. Membership of the ISHG includes participation in community activities that helps share health information and perform group physical exercises.

### Strength and limitation

Highlighting the burden of co-occurrence of multiple behavioural risk factors and its determinants among 40-year and above aged Myanmar adults in the study area is the greatest strength of this study. In addition, the evidence obtained from the current research provides updated knowledge and assistance for policy makers and healthcare providers to extend quality NCD prevention and control packages, across the nation, including strategies directed towards multiple behavioural risk factors.

Nonetheless, the present study has some limitations. The cross-sectional nature restricts the ability to draw cause-effect relationships between the potential predictors and co-occurrence of behavioural risks. Since the participants self-reported past experience and practice of behavioural risk factors, they may have elicited information bias. Moreover, differences in operational definition for co-occurrence of the risk factors, and statistical analyses also hinders the direct comparison with findings from other studies.

## Conclusions and recommendations

The current study reported significantly high prevalence of co-occurrence of behavioural risk factors among Myanmar adults in the study area. Among the four common behavioural risks for NCD, inadequate consumption of fruits and vegetables contributed the highest proportion with insufficient physical activity, the second highest. Urban residents, men, participants without formal schooling and unemployed persons were at a greater risk of co-occurrence of behavioural risk factors. Based on the evidence generated in this study, NCD prevention and control programs emphasizing management of behavioural risks should be intensified. In the regards, an awareness-raising program highlighting the importance of lifestyle modification is essential for adoption of healthy lifestyle behaviours; it is urgently needed to support health care providers in provision of essential and updated health information concerning healthy lifestyle behaviours and for implementing effective interventions directed towards multiple risk factors, and not emphasized on individual factors only. Moreover, there should be a differentiated approach in implementing awareness raising program activities in order to ensure the health education package more focused in certain target groups, rather than “one size fits all”. Further studies focusing on impact of co-occurrence and interaction of NCD risk factors and exploring the effective, culturally accepted and community-based interventions to control the behavioural risk factors should be conducted.

## Data Availability

Zenodo: Co-occurrence of behavioural risk factors for non-communicable diseases among 40-year and above aged community members in three regions of Myanmar. https://doi.org/10.5281/zenodo.7822914 ^13^. Zenodo: Questionnaire: Co-occurrence of behavioural risk factors for non-communicable diseases among 40-year and above aged community members in three regions of Myanmar. https://doi.org/10.5281/zenodo.7877944 ^14^. Data are available under the terms of the Creative Commons Attribution 4.0 International license (CC-BY 4.0).
