# Supplementary material for: Co-occurrence of behavioural risk factors for non-communicable diseases among 40-year and above aged community members in three regions of Myanmar
Source: Open Res Eur. 2024 Jan 11;3:77. Originally published 2023 May 15. [Version 2] doi: 10.12688/openreseurope.15859.2 (PMC10864818; doi:10.12688/openreseurope.15859.2)
Supplement: Peer review history

# Reviewer report: Reviewer response for version 2

**DOI:** 10.21956/openreseurope.18458.r37406

**Published:** 2024-02-13

**Authors:** Thirunavukkarasu Sathish

**Affiliations:** 1 Emory University, Atlanta, Georgia, USA

No further comments to make.

Is the study design appropriate and does the work have academic merit?

Yes

Is the work clearly and accurately presented and does it cite the current literature?

Yes

If applicable, is the statistical analysis and its interpretation appropriate?

Yes

Are all the source data underlying the results available to ensure full reproducibility?

Yes

Are the conclusions drawn adequately supported by the results?

Yes

Are sufficient details of methods and analysis provided to allow replication by others?

Yes

Reviewer Expertise:

Chronic diabetes epidemiology, type 2 diabetes prevention, implementation science, lifestyle interventions

I confirm that I have read this submission and believe that I have an appropriate level of expertise to confirm that it is of an acceptable scientific standard.

# Reviewer report: Reviewer response for version 1

**DOI:** 10.21956/openreseurope.17132.r32199

**Published:** 2023-08-22

**Authors:** Thirunavukkarasu Sathish

**Affiliations:** 1 Emory University, Atlanta, Georgia, USA

This study looked at the clustering of health behaviors for chronic diseases among adults aged 40 years and above in Myanmar. Multimorbidity is an important topic that has gained significant attention in recent years. Overall, the manuscript is well-written, with some potential policy implications for the healthcare system in Myanmar. 

 My specific comments:

1. Justify why 40 years was used as a cut-off.
2. Consider presenting the sample selection procedure as a figure. 
3. How was a sample size of 660 calculated?
4. The WHO STEPS survey defines current smokers as those who smoked at least one tobacco product in the last 30 days. Please justify your definition of current smokers. 
5. Tables: Provide n along with % for each variable. 
6. Cut down the text of the first paragraph of the discussion section. It is too long!
7. "Highlighting the burden of co-occurrence of multiple behavioural risk factors and its determinants among 40-year and above aged Myanmar adults in the study area is the greatest strength of this study". This is the study objective. Not clear how this is a strength of the study?
8. "Moreover, differences in operational definition for co-occurrence of the risk factors, and statistical analyses also hinders the direct comparison with findings from other studies". This is not a limitation. Please remove it. 
9. Please list more relevant limitations. 
10. Cut down the text of the conclusion. Be concise and to the point.

Is the study design appropriate and does the work have academic merit?

Yes

Is the work clearly and accurately presented and does it cite the current literature?

Yes

If applicable, is the statistical analysis and its interpretation appropriate?

Yes

Are all the source data underlying the results available to ensure full reproducibility?

Yes

Are the conclusions drawn adequately supported by the results?

Yes

Are sufficient details of methods and analysis provided to allow replication by others?

Yes

Reviewer Expertise:

Chronic diabetes epidemiology, type 2 diabetes prevention, implementation science, lifestyle interventions

I confirm that I have read this submission and believe that I have an appropriate level of expertise to confirm that it is of an acceptable scientific standard, however I have significant reservations, as outlined above.

# Response

**Published:** 2024-01-11

**Authors:** Aye Sandar Mon

**Affiliations:** Department of Biostatistics and Medical Demography, University of Public Health, Yangon, Myanmar, Yangon, Yangon, Myanmar

Thank you for your expert opinion and invaluable suggestions. Below are the responses to each review point and question:  1. Justify why 40 years was used as a cut-off. This study was conducted on the data included as part of the SUNISEA project that aims to strengthen basic health services, scale up strategies for non-communicable disease prevention and control, and strengthen synergy between primary healthcare providers and communities for the prevention and control of NCDs. Therefore, during the project period, the running NCD prevention and control activities in Myanmar have been implemented through PEN (WHO package of essential NCD interventions) clinics which targeted at 40-year and above aged population. Another reason is, since SUNISEA project aims at assessing synergy between primary healthcare providers and community group, the inclusive self-help group (ISHG), one of the community groups, covers the older aged group. Due to the two reasons mentioned above, the study units were targeted at 40-year and above aged people.  2. Consider presenting the sample selection procedure as a figure.  The figure for sampling procedure has been added in the revised version (version 2) of the article.

   3. How was a sample size of 660 calculated? For this study, we utilized cross-sectional survey data collected during the baseline measurement in the retrospective phase of the SUNI-SEA project. One of the baseline survey objectives was to assess the proportion of raised blood glucose. The sample size was calculated using the formula for “estimating a population proportion with a specified relative precision” (Lemeshow et al., 1990). The estimated proportion “P” was set at 20% for individuals diagnosed with pre-diabetes or diabetes according to the STEP 2014 report, with a confidence level of 95% and a relative precision error (ε) set at 20%. After considering the design effect (1.5) due to the multistage sampling design and accounting for a 10% non-response rate, the minimum required sample size was determined to be “660”. Since the current study analyzed only a portion of the baseline data and its specific objective did not involve sample size determination, we have not provided detailed information on sample size calculation in this manuscript. Reference Lemeshow, S., David W. Hosmer, J., Klar, J., & Lwanga, S. K. (1990). Adequacy of sample size in health studies. Wiley.    

   4. The WHO STEPS survey defines current smokers as those who smoked at least one tobacco product in the last 30 days. Please justify your definition of current smokers. 

 In this study, smoking was considered when anyone currently smokes any tobacco products. This behavioral risk was operationally defined, and its prevalence was calculated based on a question from the study questionnaire: 'Do you currently smoke any tobacco products, such as cigarettes, cigars, or pipes?' (Using Showcard). While this operational definition applied to the term “smoking”, it was not explicitly used for the designation “current smokers”.   5. Tables: Provide n along with % for each variable. 

  “n” has been provided with % for each variable intable 2 and 3 in revised version. However, in table 1, the proportion of the polytomous outcome variable (co-occurrence of behavioural risk factors) was presented together with 95% CI across age and gender strata and thus, if “n” is presented with % for each stratum (in addition to total frequency), there will be information overload for descriptive table.   6. Cut down the text of the first paragraph of the discussion section. It is too long! This paragraph has been revised as per your suggestion.

   7. "Highlighting the burden of co-occurrence of multiple behavioural risk factors and its determinants among 40-year and above aged Myanmar adults in the study area is the greatest strength of this study". This is the study objective. Not clear how this is a strength of the study? To date, the study assessing the co-occurrence of behavioural risk factors and its determinants among the study population has been the first study in this study area and the results can be applied to the expansion of NCD prevention and control program in this area. Therefore, we consider this as the greatest strength of our study.  

   8. "Moreover, differences in operational definition for co-occurrence of the risk factors, and statistical analyses also hinders the direct comparison with findings from other studies". This is not a limitation. Please remove it.  This has been already removed in the revised version. 

   9. Please list more relevant limitations.  Lack of temporal sequence between exposure and outcome due to the cross-sectional study and information bias due to the self-reported experience of behavioural risk factors were considered as the limitations of this study. Moreover, limited generalizability and limited scope of behavioural risk factors have been added in the revised version. 

   10. Cut down the text of the conclusion. Be concise and to the point. In the conclusion section, we present both the conclusion and recommendations. Following your suggestion, we revised the section by removing certain points. In the revised version, only the first four lines encapsulate the conclusion, while the remaining lines focus on recommendations.

# Reviewer report: Reviewer response for version 1

**DOI:** 10.21956/openreseurope.17132.r31647

**Published:** 2023-06-01

**Authors:** May Soe Aung

**Affiliations:** 1 Department of Preventive and Social Medicine, University of Medicine 1, Yangon, Yangon, Myanmar

Thank you for an opportunity to review such an interesting manuscript that highlights the research gap regarding non-communicable diseases in Myanmar. The aim of study is well justified and statistical analysis is sound to reach the relevant findings. Some following remarks are added for more completeness of the manuscript.

1. In working definitions, it is better to mention how primary and secondary level of education are classified.
2. Authors are suggested to add detail explanation about assessing the knowledge on risk factors for hypertension and diabetes mellitus.
3. The outcome variable, co-occurrence of behavioural risk factors for NCD categorized into three groups should be displayed by a figure for more clear presentation.
4. It is interested to know the purpose of Table (1) that describes the outcome for only age and sex structure and why the age groups are shown by separate sex. The data about age groups under total (N=660) is repeatedly found in Table (3) as association with outcome. Therefore, it should be considered whether table (1) is necessary for the manuscript.
5. In figure 1, the word “physical” is needed to add between “insufficient” and “activity”.
6. According to multinomial logistic regression, it is concluded that urban residents, men, participants without formal schooling and unemployed persons were at a greater risk of co-occurrence of behavioural risk factors. Based on this finding, the recommendation will be better by emphasizing on each of these groups rather than consideration generally. Therefore, authors are kindly suggested to provide specific comments for urban residents, men, participants without formal schooling and unemployed persons to control the behavioural risk factors among them.
7. The last but not the least is that the declaration about any potential or perceived competing interests about the research related works is important to add in the manuscript.

Is the study design appropriate and does the work have academic merit?

Yes

Is the work clearly and accurately presented and does it cite the current literature?

Yes

If applicable, is the statistical analysis and its interpretation appropriate?

Yes

Are all the source data underlying the results available to ensure full reproducibility?

Yes

Are the conclusions drawn adequately supported by the results?

Yes

Are sufficient details of methods and analysis provided to allow replication by others?

Yes

Reviewer Expertise:

I have some research experiences related to NCD risk factors in both urban and rural areas of Myanmar. I also have involved in Global NCD Risk Factor Collaboration (NCD-RisC) from 2016 up to 2023.

I confirm that I have read this submission and believe that I have an appropriate level of expertise to confirm that it is of an acceptable scientific standard.

# Response

**Published:** 2024-01-11

**Authors:** Aye Sandar Mon

**Affiliations:** Department of Biostatistics and Medical Demography, University of Public Health, Yangon, Myanmar, Yangon, Yangon, Myanmar

Thank you for your expert opinion and invaluable suggestions. The following are my responses to each review point and question: 1. In working definitions, it is better to mention how primary and secondary level of education are classified. The categorization of education together with distribution of the proportion of the outcome in each category has been mentioned in the result section.

   2. Authors are suggested to add detail explanation about assessing the knowledge on risk factors for hypertension and diabetes mellitus. The explanation has been added in the revised version (version 2).

   3. The outcome variable, co-occurrence of behavioural risk factors for NCD categorized into three groups should be displayed by a figure for more clear presentation. This information has been mentioned in table 1 and therefore, in order to avoid the duplication of information, the figure regarding the outcome variable was not presented.   4. It is interested to know the purpose of Table (1) that describes the outcome for only age and sex structure and why the age groups are shown by separate sex. The data about age groups under total (N=660) is repeatedly found in Table (3) as association with outcome. Therefore, it should be considered whether table (1) is necessary for the manuscript. In Table 1, the proportion of co-occurrence of behavioral risk factors, along with 95% CI, was presented across age and sex strata. The table reveals an increasing pattern of co-occurrence of three or more risks with age in females. Therefore, we include this table.  

   5. In figure 1, the word “physical” is needed to add between “insufficient” and “activity”. The term has been revised in the version 2.

   6. According to multinomial logistic regression, it is concluded that urban residents, men, participants without formal schooling and unemployed persons were at a greater risk of co-occurrence of behavioural risk factors. Based on this finding, the recommendation will be better by emphasizing on each of these groups rather than consideration generally. Therefore, authors are kindly suggested to provide specific comments for urban residents, men, participants without formal schooling and unemployed persons to control the behavioural risk factors among them. We have mentioned a recommendation in line with your suggestion: “there should be a differentiated approach in implementing awareness raising program activities in order to ensure the health education package more focused in certain target groups, rather than “one size fits all”.”   

   7. The last but not the least is that the declaration about any potential or perceived competing interests about the research related works is important to add in the manuscript. This declaration has already been included in the first version as “No competing interests were disclosed”.
